# Whole-Genome Assembly of Yersinia pestis 231, the Russian Reference Strain for Testing Plague Vaccine Protection

**DOI:** 10.1128/MRA.01373-20

**Published:** 2021-02-04

**Authors:** Angelina A. Kislichkina, Ekaterina A. Krasil’nikova, Mikhail E. Platonov, Yury P. Skryabin, Angelika A. Sizova, Viktor I. Solomentsev, Tat’yana V. Gapel’chenkova, Svetlana V. Dentovskaya, Alexandr G. Bogun, Andrey P. Anisimov

**Affiliations:** a State Research Center for Applied Microbiology and Biotechnology, Obolensk, Russia; Broad Institute

## Abstract

We report the whole-genome sequence of Yersinia pestis subsp. *pestis* bv. Antiqua strain 231 belonging to the 0.ANT3 phylogroup, the reference strain for testing plague vaccine protection in Russia. Genome sequencing was completed using the Oxford Nanopore MinION and Illumina platforms.

## ANNOUNCEMENT

Yersinia pestis is a Gram-negative bacterium responsible for the three plague pandemics that have claimed more than 200 million lives (1). Antibiotic therapy is effective in treating plague, but recently, strains resistant to all antimicrobial remedies recommended by WHO experts have been increasingly isolated (2). A good alternative to antibiotics is the plague vaccine, which is still being developed in many laboratories. To obtain comparable results and to avoid discrepant conclusions in them, it is necessary to agree on the same standard operating procedures using the same strains for testing virulence and plague vaccines. However, taking into account the practical impossibility of interstate exchange of Yersinia pestis strains, it would be reasonable at least to use the strains with the most comprehensive characterization, including the whole-genome sequencing. Here, we report the whole-genome sequence of Y. pestis strain 231 (no. SCPM-O-B-6899 in the culture collection of the State Research Center for Applied Microbiology and Biotechnology), maintained in Russian plague laboratories since 1947 after isolation from a marmot in Kyrgyzstan.

The bacteria were cultured on brain heart infusion agar (HiMedia, India) for 18 h at 28°C, and DNA samples were extracted using a DNA minikit (BioFact, Daejeon, Republic of Korea).

Genome sequencing was completed using the Oxford Nanopore MinION and Illumina platforms. For the Illumina MiSeq instrument, DNA libraries were prepared using a Nextera DNA library preparation kit and a MiSeq reagent kit v3 (300 cycles). Whole-genome sequencing was performed following the manufacturer’s protocols. For Oxford Nanopore MinION sequencing, we used a rapid barcoding kit (RBK004) and MinION flow cell (R9.4.1). Whole-genome sequencing was performed with the MinKNOW software v19.05.0 (time, 48 hours; 180 mV), and base calling was performed using Guppy v3.1.5.

Up to 805,206 paired-end reads (218,742,162 bases) with a coverage depth of 47-fold were generated by the Illumina system, and 90,258 long single reads with an average length of 14,800 bp (total count of 1,335,879,124 bp) were generated by the MinION system. The hybrid genome was assembled from all reads without primary filtering using the software Unicycler v0.4.7 with default settings (3). The resulting assembly included four circular contigs. The contigs were classified as a chromosome or plasmids using BLAST (nucleotide search).

The final genome assembly contained one chromosome and three plasmids, and the genome contained 4,806,721 bp with a 47.61% G+C content. In detail, the chromosome has 4,625,829 bp, plasmid pCD has 70,303 bp, plasmid pMT has 100,979 bp, and plasmid pPCP has 9,610 bp. The final genome was annotated with the NCBI Prokaryotic Genome Annotation Pipeline (4). This strain has a total of 4,549 genes, which are composed of 4,253 protein coding sequences (CDSs), 107 RNA genes, and 189 pseudogenes.

This study will facilitate the understanding of microbial characteristics of Y. pestis 231 and will provide an opportunity for its comparison with other strains for testing virulence and plague vaccines of the plague pathogen.

### Data availability.

The genome sequences for Y. pestis 231 have been deposited in GenBank under accession no. CP045145.1 for the chromosome and CP045146.1, CP045147.1, and CP045148.1 for the plasmids. The raw sequence reads have been deposited in the SRA under accession no. SRR10259785 (MiSeq reads) and SRR10259784 (MinION reads).
